# Assessing weight perception accuracy to promote weight loss among U.S. female adolescents: A secondary analysis

**DOI:** 10.1186/1471-2458-10-465

**Published:** 2010-08-09

**Authors:** Jennifer Yost, Barbara Krainovich-Miller, Wendy Budin, Robert Norman

**Affiliations:** 1 School of Nursing, McMaster University, Hamilton, Ontario, CA, USA; 2 College of Nursing, New York University, New York, NY, USA; 3 NYU Langone Medical Center, New York, NY, USA and College of Nursing, New York University, New York, NY, USA; 4 Department of Epidemiology &Health Promotion Director of Biostatistics, Bluestone Center for Clinical Research Colleges of Dentistry & Nursing, New York University, New York, NY, USA

## Abstract

**Background:**

Overweight and obesity have become a global epidemic. The prevalence of overweight and obesity among U.S. adolescents has almost tripled in the last 30 years. Results from recent systematic reviews demonstrate that no single, particular intervention or strategy successfully assists overweight or obese adolescents in losing weight. An understanding of factors that influence healthy weight-loss behaviors among overweight and obese female adolescents promotes effective, multi-component weight-loss interventions. There is limited evidence demonstrating associations between demographic variables, body-mass index, and weight perception among female adolescents trying to lose weight. There is also a lack of previous studies examining the association of the accuracy of female adolescents' weight perception with their efforts to lose weight. This study, therefore, examined the associations of body-mass index, weight perception, and weight-perception accuracy with trying to lose weight and engaging in exercise as a weight-loss method among a representative sample of U.S. female adolescents.

**Methods:**

A nonexperimental, descriptive, comparative secondary analysis design was conducted using data from Wave II (1996) of the National Longitudinal Study of Adolescent Health (Add Health). Data representative of U.S. female adolescents (N = 2216) were analyzed using STATA statistical software. Descriptive statistics and survey weight logistic regression were performed to determine if demographic and independent (body-mass index, weight perception, and weight perception accuracy) variables were associated with trying to lose weight and engaging in exercise as a weight-loss method.

**Results:**

Age, Black or African American race, body-mass index, weight perception, and weight perceptions accuracy were consistently associated with the likeliness of trying to lose weight among U.S. female adolescents. Age, body-mass index, weight perception, and weight-perception accuracy were positively associated (*p *< 0.05) with trying to lose weight. Black/African American subjects were significantly less likely than their White counterparts to be trying to lose weight. There was no association between demographic or independent variables and engaging in exercise as a weight-loss method.

**Conclusions:**

Findings suggest that factors influencing weight-loss efforts, including age, race, body-mass index, weight perception, and weight-perception accuracy, should be incorporated into existing or new multi-component weight-loss interventions for U.S. adolescent females in order to help reduce the national epidemic of overweight and obesity among U.S. female adolescents.

## Background

Due to the increased worldwide prevalence of overweight and obesity, health care officials consider overweight and obesity a global epidemic [1,2], especially among young people. The latest estimates among children and adolescents in 34 countries indicate a prevalence of overweight ranging from 5.1% to 25.4% and obesity from 0.4% to 7.9%. Overweight and obesity also represent a national epidemic in the United States, where the prevalence of overweight and obesity has almost tripled among children and adolescents in the last 30 years. Data from the National Health and Nutrition Examination Survey indicate the prevalence of overweight U.S. adolescents (12 to 19 years old) increased from 6.1% in 1971-1974 to 18% in 2005-2006, placing the United States second among countries with the highest incidence of overweight and obesity (25.1%) [1,3]

Under the influence of numerous factors, overweight and obesity develop from an imbalance between energy intake and expenditure [4,5]. Overweight and obesity in childhood and adolescence can lead to consequences extending into adulthood, such as type 2 diabetes, hypertension, atherosclerosis, and poor quality of life [4]. To avoid such consequences, developing effective interventions for overweight and obese children and adolescents has become a major public health issue [6]. Results from recent systematic reviews demonstrate that no single, particular intervention or strategy successfully assists overweight or obese adolescents in losing weight [7,8]. Instead, researchers suggest that effective interventions for adolescents consist of multiple components, including behaviour skills, behaviour change, and parental involvement to promote healthy diet and nutrition and to increase physical activity (PA)/exercise [7-9].

Despite recommendations for healthy weight-loss behaviours, adolescents engage in numerous unhealthy weight-loss behaviours [10], such as taking diet pills, laxatives, and diuretics, employing self-induced vomiting, and skipping meals [11-14]. Evidence suggests that female adolescents engage in unhealthy weight-loss behaviours more frequently than do male adolescents [11,13]. In addition, unhealthy weight-loss behaviours are more likely to occur among overweight or obese female adolescents [15,16] and among female adolescents who perceive themselves to be overweight or obese [17,18]. Consequently, female adolescents need effective, multi-component, weight-loss interventions that promote healthy weight-loss behaviours.

An increased understanding of factors that influence female adolescents' desire to lose weight and their choice of health-promoting weight-loss behaviours will help develop more effective weight-loss interventions. Therefore, the purpose of the present study was to determine whether body-mass index (BMI) and weight perception are associated with trying to lose weight and engaging in exercise as a weight-loss method among U.S. female adolescents.

### Review of the Literature

The following literature review was conducted in order to identify what is known and not known about the variables under consideration in this secondary analysis study. The strengths of previously conducted studies considering similar variables included the use of large probability samples representative of U.S. and international populations of adolescents, objective measurements of height and weight, and consideration of associations between variables and trying to lose weight among female adolescents. However, these previous relevant studies also included the following limitations: convenience samples that limited generalizability beyond the studies' population; participants' self-reports of height and weight; and the researchers' use of tools with insufficiently reported and/or lack of validity and reliability, which undermined external and internal validity and included various classifications of actual weight (BMI) and weight perception. Considering such limitations and in an effort to improve the ability to synthesize previous findings, only studies that used probability-sampling methods were included in the following literature review.

Researchers report a significant percentage of adolescents are trying to lose weight, ranging from approximately 34% to 42% in the United States [16,19] to 61% in Quebec, Canada [20]. The most consistent demographic finding is that female adolescents in the United States [15,21,22] and worldwide [22-24] are significantly more likely to be trying to lose weight than are their male counterparts. For example, Ojala *et al. *[22]. estimated that, among their sample of adolescents from over 30 countries, 19.8% female adolescents were trying to lose weight compared to 7.5% male adolescents in their cross-national 2007 survey.

A limited number of studies have considered demographic variables other than nationality, such as race/ethnicity, age, and socioeconomic status (SES), among adolescents trying to lose weight. Research findings of U.S. adolescents demonstrate either that no relationship exists between race/ethnicity and trying to lose weight [21] or that Black/African American female adolescents are less likely to be trying to lose weight than their Hispanic and White/non-Hispanic White counterparts [11,25]. In addition, study findings show a significant positive relationship between age and trying to lose weight in U.S. adolescents [26] and worldwide [22], in that older adolescents are more likely to be trying to lose weight. Lastly, among demographic variables in a sample of female adolescents from five major U.S. cities, researchers found no evidence of a relationship between SES (as measured by parental education) and trying to control or lose weight [21].

Previous study findings indicate BMI and weight perception are associated with obese and overweight adolescents' efforts to lose weight. Researchers report that a significant positive association exists between trying to lose weight and BMI among adolescents in the United States and internationally [20,22,23,27,28]. Furthermore, findings indicate that obese female adolescents are significantly more likely to be trying to lose weight than overweight female adolescents [22]. Researchers also report that a relatively large percentage of U.S. adolescents who perceive themselves as overweight are trying to lose weight (70.3%) [26], with greater percentages of White/non-Hispanic White adolescent females who perceive themselves as "too fat" trying to lose weight, compared to Hispanic and Black/African American adolescents [16]. These data and associations are limited, however, as the only significant positive association between weight perception and trying to lose weight was reported among a sample of adolescents in Beirut, Lebanon [23]. However, again, the finding is limited due to its lack of generalizability.

Studies present conflicting data on whether U.S. adolescent females are significantly more likely [11,26,29] or less likely [15,25] than U.S. adolescent males to exercise as a means to control and/or lose weight. Nevertheless, study findings indicate exercise is the most common behaviour among U.S. and international female adolescents trying to control or lose weight [22,28]. Researchers also report that African American [15,25] and Hispanic [25] female adolescents in the United States are significantly less likely to exercise than their White counterparts. Although no studies have considered the association between adolescents' weight perception and engaging in exercise to trying to lose weight, researchers report a significant positive relationship between BMI and engaging in exercise to trying to lose weight among U.S. female adolescents [25].

Thus, based on findings reported in the literature, female adolescents are more likely than male adolescents to be trying to lose weight and are most likely to engage in exercise to do so. However, limited evidence demonstrates associations between demographic variables, BMI, and weight perception among female adolescents trying to lose weight. Furthermore, previous studies have not examined the association of the accuracy of female adolescents' weight perception with their efforts to lose weight. To fill this gap in the literature, the current study focused on identifying whether BMI and weight perception are associated with trying to lose weight and engaging in exercise as a weight-loss method among U.S. female adolescents. This study addressed the following research questions: (a) Are BMI and weight perception associated with trying to lose weight among female adolescents, controlling for age, race/ethnicity, and SES?; and (b) Are BMI, weight perception, and trying to lose weight associated with female adolescents' report of engaging in exercise as a weight-loss method, controlling for age, race/ethnicity, and SES?

## Methods

### Design

The current study was a nonexperimental, descriptive, comparatory secondary analysis of data from the National Longitudinal Study of Adolescent Health [30]. n.d.a). Add Health was a longitudinal, school-based study that collected data on a variety of health-related behaviours in a nationally representative sample of U.S. adolescents in grades 7 through 12. Using a multistage, stratified, school-based cluster sampling design, Add Health subjects were first enrolled during the administration of an in-school questionnaire in 1994 (Wave I, Stage 1). One year later, additional subjects from school rosters were enrolled during in-home interviews (Wave 1, Stage 2; *N *= 20,745). Add Health subjects were subsequently followed over time during in-home interviews 2 and 6 years later: Wave II (1996; *N *= 14,738) and Wave III (2000; *N *= 15,170) [30].

### Sample for the Present Study

The present study included females aged 13 to 18 years old on whom data were collected during in-home interviews at Wave I and Wave II of the Add Health study. Female subjects were excluded if they (a) were not included in the core sample; (b) were younger than 13 years or older than 18 years at Wave II; (c) were missing data for age, race/ethnicity, or SES; (d) reported pregnancy at Wave II; and (e) were determined to be physically disabled during data collected at Wave II. The Add Health public-use data set provides data for more than 2,000 female adolescents at Wave II who met inclusion criteria. Due to the large sample available for the present study, a power analysis was deemed unnecessary upon statistical consultation.

### Data Collection

Data for the present study were obtained from data collected from the Add Health study. From April to August 1996, highly structured in-home interviews were conducted by trained interviewers using computer-assisted self-interview for Add Health Wave II data collection [31]. All measures were self-reported, except for height and weight, which were measured by the trained interviewers immediately following the interview. Demographic data for the current study were obtained from the Add Health Wave I in-home interviews (Stage 2), which followed the same data-collection procedures as described for Wave II. Although further information is available from the Add Health Web site http://www.cpc.unc.edu/projects/addhealth, Add Health-sponsored publications lack information regarding the validity and reliability of the study's in-home interviews. In addition, recommended techniques were not available in the current secondary analysis study [32].

### Measures

#### Demographic Variables

Each subject's age was calculated by subtracting the subject's date of birth from the date of data collection recorded on the laptop computer used for data collection. Subjects ethnicity was identified as Hispanic or Latino or not Hispanic or Latino. Subjects also self-identified themselves into one of the following race categories: White, Black or African American, American Indian or Native American, or Asian or Pacific Islander.

For the current study, subjects' socioeconomic status was operationalized to include two separate measures. Research on health-disparities lacks accepted measures of socioeconomic status [33-35]. Although single and composite measures exist, evidence suggests that occupation may not be a useful indicator of adolescents' SES and that educational attainment, which is not interchangeable with family income, is the most widely used indicator of adolescents' SES [33,35]. Therefore, in the current study, the two operationalized measures for SES were highest maternal educational attainment and family income [1994 household total income (in thousands of dollars) before-tax and household income received in 1994 (including income from all household members, dividends, welfare benefits, and other sources)].

#### Body-Mass Index

The measure used for actual weight, BMI, was calculated as weight (in kilograms) divided by height (in meters) [kg/m^2^]. Although standardized classification systems of overweight and obesity for adolescents do not exist, classifying adolescents' weight based on age- and gender-specific BMI cut-off points is accepted worldwide [36]. In the present study, classifications from the National Health and Nutrition Examination Survey were used to identify female adolescents' BMI for age and gender as obese (> 95^th ^percentile), overweight (85^th ^to 95^th ^percentile), or normal weight (< 85^th ^percentile). Dichotomous variables were created to identify subjects as obese or overweight.

#### Weight Perception

As an important perceptual dimension of body image, weight perception is defined as the perception of one's body weight [37]. In the Add Health Wave II in-home interviews, subjects' weight perception was assessed by the question, "How do you think of yourself in terms of weight?" Response choices included "very underweight," "slightly underweight," "about the right weight," "slightly overweight," or "very overweight." The responses of "very underweight," "slightly underweight," and "about the right weight" were collapsed into a single "not overweight" response of weight perception.

#### Accuracy of Weight Perception

To measure the accuracy of subjects' weight perception, each subject's BMI and weight perception were compared. From this comparison, four variables were created. The first variable (reference category) indicated subjects who were not overweight or obese and perceived themselves in any of the weight perception categories (very underweight, slightly underweight, about the right weight, slightly overweight, or very overweight). The second variable indicated overweight or obese subjects who underestimated their weight. Despite being overweight or obese, these subjects did not perceive themselves as overweight (weight perception was very underweight, slightly underweight, or about the right weight (*Overweight or Obese, Not Overweight weight perception*). The third and fourth variables indicated subjects with a degree of accuracy in their weight perception. These subjects were overweight or obese and perceived themselves as either slightly overweight (*Overweight or Obese, Slightly Overweight weight perception*) or very overweight (*Overweight or Obese, Very Overweight weight perception*) (Table 1). Due to the small number of female subjects who were obese and perceived themselves very overweight (*n *= 88), it was not possible to construct a separate weight perception accuracy variable for subjects whose BMI percentile indicated they were obese.

**Table 1:** Classification of Weight Perception Accuracy

	Weight Perception		
	
BMI Classification	Not Overweight	Slightly overweight	Very overweight
Overweight or Obese	Inaccurate Underestimation	Accurate Estimation	Accurate Estimation

#### Trying to Lose Weight

To determine whether subjects were currently trying to lose weight, responses to the question, "Are you trying to lose weight, gain weight, or stay the same weight?" were examined. To create a variable that identified subjects who were currently trying to lose weight, the responses "gain weight" and "stay the same weight" were collapsed together into "not trying to lose weight."

#### Engaging in Exercise as a Weight-Loss Method

An aim of this study was to determine whether BMI and weight perception are associated with trying to lose weight and reports of engaging in exercise as a weight-loss method among U.S. female adolescents. Upon determining subjects were trying to lose weight, they were identified as engaging in exercise to try to lose weight if they answered "yes" to the "Exercise" response to the question, "During the past seven days, which of the following did you do in order to lose weight or keep from gaining weight?"

### Data Analysis

Data were analyzed using STATA, a statistical software package, to account for the Add Health study's cluster sampling with unequal probability design and using Add Health sampling weights for cross-sectional analyses, which allowed for a nationally representative sample of U.S. female adolescents [38]. Results were considered statistically significant if the *p*-value was less than of 0.05. Initial analyses included descriptive statistics for sample characteristics and a summary statistics of variables. Chi square statistics determined relationships between dichotomous variables. After determining the presence of low to modest correlations between all independent variables (ranging from -0.2232 to 0.4889) in each model, survey-weighted logistic regression was performed to determine the effect of a dichotomous or continuous independent variable on the probability of a dichotomous dependent variable. Wald tests, when appropriate, were performed to determine the significance between the independent variable and dependent variable [39]. For each of the current study's research questions, three models were used to predict the dependent variable. The first model contained only demographic variables. The second model contained demographic variables as well as BMI and weight perception as independent variables. The third model contained demographic variables and variables indicating subjects' weight perception accuracy as independent variables.

## Results

### Sample Description

Of the 2,510 subjects in the Add Health public-use data core sample, a total of 2,216 subjects were eligible for secondary analysis in the current study, after applying inclusion and exclusion criteria. Subjects were 13 to 18 years old (*M *= 15.8, *SD *= 1.5), with the majority between 15 and 17 years old. As shown in Table 2, which depicts the sample's demographic variables, most subjects were non-Hispanic (88%) and White (69%); however, other ethnic (Hispanic) and race categories (Black or African American, American Indian or Native American, and Asian or Pacific Islander) were also represented in the sample. Although 85.1% of the subjects' mothers reported having graduated from high school or earned a General Equivalency Diploma, this percentage was slightly lower than the 1994 percentage (91.6%) of educational achievement rates for a high school diploma or greater in the U.S. for females 24 to 64 years old [40]. In addition, the subjects' median total household income ($40,000) was slightly higher than that of U.S. households in 1994 ($32,264) [41].

**Table 2:** Descriptive Statistics for Demographic Variables (N = 2216)

Variable (n)	n	%
Age at Wave II (2216)		
Mean (Standard Deviation)	15.80	(1.46)
Ethnicity (2208)		
Hispanic	263	11.91
non-Hispanic	1945	88.09
Race (2210)		
White	1531	69.28
Black or African American	425	19.23
American Indian or Native American	44	1.99
Asian or Pacific Islander	73	3.30
Maternal Educational Attainment (1977)		
8^th ^grade or less	90	4.55
> 8^th ^grade, not high school graduate	193	9.76
Business/trade, not high school graduate	11	0.56
High school graduate	560	28.33
Completed a General Equivalency Diploma	94	4.75
Business/trade after HS	187	9.46
Non-college graduate	359	18.16
College graduate	282	14.26
Post-graduate	201	10.17
Total Household Income (1742)^a^		
Mean (Standard Deviation)	48.90	(56.5)
Median	40.00	
Range	0-900	

*Note*. ^a ^in thousands of dollars

### Weight Related Variables

#### Body-Mass Index

The majority of female adolescents in this study sample were not overweight or obese (72.63%) (see Table 3). However, significant racial differences existed in the sample's weight status. Black/African American and American Indian/Native American female adolescents had the highest prevalence of overweight and obesity. Chi-square statistics indicated Black/African American female adolescents were significantly more likely to be overweight or obese than female adolescents who were White, Asian/Pacific Islander, or in the race category of "Other" (*p *< 0.01). American Indian/Native American female adolescents were significantly more likely to be overweight or obese than White (*p *< 0.05) or Asian/Pacific Islander (*p *< 0.01) female adolescents. Asian/Pacific Islander female adolescents were the least likely to be overweight compared to female adolescents in all other racial categories (*p *< 0.01).

**Table 3:** Descriptive Statistics for Body Mass Index (BMI) Percentile, Weight Perception, Trying to Lose Weight, and Exercise as a Weight-Loss Method (N = 2216)

Variables (n)	n	%
BMI Percentile (2163)		
Mean (Standard Deviation)	22.90	(5.18)
Median	21.63	
Not overweight or obese	1571	72.63
Overweight	336	15.53
Obese	256	11.84
Weight Perception (2215)		
Not overweight	1351	60.99
Slightly overweight	757	34.18
Very overweight	107	4.83
Trying to Lose Weight at Wave II (2215)		
Not trying to lose weight	1,175	53.05
Trying to lose weight	1,040	46.95
Exercise as a Weight-Loss Method if Trying to Lose Weight (1040)		
No	244	23.46
Yes	796	76.54

#### Weight Perception

In the study's sample, most female adolescents perceived themselves as not overweight (60.99%) (see Table 3). American Indian/Native American female adolescents and female adolescents in the "Other" race category were most likely to perceive themselves as overweight. Chi-square statistics demonstrated that American Indian/Native American and "Other" female adolescents were significantly more likely than Black/African American female adolescents to perceive themselves as overweight (*p *< 0.05). Subjects in the "Other" race category were also significantly more likely than their White counterparts to perceive themselves as overweight (*p *< 0.05).

#### Accuracy of Weight Perception

In addition to descriptive statistics, chi square statistics demonstrated a significant difference in the accuracy of female adolescents' weight perception, based on their actual BMI weight classification. Of the female adolescents who were overweight or obese, 79.06% accurately perceived themselves as either slightly or very overweight (*p *< 0.05). In addition, almost 20% of the overweight or obese female adolescents inaccurately underestimated their weight as not overweight (see Table 4).

**Table 4:** Cross Tabulations for Weight Perception Accuracy^a ^(N = 2163)

	Weight Perception		
		
BMI Percentile	Not Overweight	Slightly or Very Overweight	
	n (%)	n (%)	n
Not Overweight or Obese (< 85^th ^percentile)	1206 (76.77%)	365 (23.23%)	1,571 (100%)
Overweight or Obese (< 85^th ^percentile)	124 (20.95%)	469 (79.05%)	592 (100%)
	
n	1,330 (61.49%)	833 (38.51%)	2,163

*Note: *^a ^chi2(1) 565.78 p < 0.01

Significant racial differences also existed in the subjects' accuracy of weight perception. Overweight or obese Black/African American female adolescents were more likely to underestimate their weight than were White (*p *< 0.01), Asian (*p *< 0.01), or Other (*p *< 0.05) female adolescents. In addition, overweight or obese American Indian/Native American female adolescents were significantly more likely than their Asian counterparts to underestimate their weight (*p *< 0.05).

#### Trying to Lose Weight

Approximately half of the female adolescents in the sample were currently trying to lose weight, while the other half indicated they were not currently trying to lose weight (see Table 3). Across all models used in the study, age was significantly associated with trying to lose weight: With each increasing year in age, subjects were more likely to be trying to lose weight (*OR *= 1.07-1.18, *p *< 0.05) (see Table 5). In the third model (demographic variables, plus variables indicating weight perception accuracy), race was significantly associated with trying to lose weight: Black/African American subjects were 0.6 times less likely to be trying to lose weight than their White counterparts. A significant positive association with also emerged between BMI and weight perception. Overweight and obese subjects were two to four times more likely to be trying to lose weight than subjects who were not overweight or obese. Also, subjects who perceived themselves as slightly or very overweight were 13 to 25 times more likely to be trying to lose weight than subjects who did not perceive themselves as slightly or very overweight (see Table 5).

**Table 5:** Results of Survey-Weighted Logistic Regression for Trying to Lose Weight

	Model 1		Model 2		Model 3	
			
Variable (reference category)	OR^a ^	CI (95%)^b^	OR^a ^	CI (95%)^b^	OR^a ^	CI (95%)^b^
Age	**1.07^c^**	**1.005-1.15**	**1.11^c^**	**1.02-1.22**	**1.18^c^**	**1.09-1.27**
Hispanic origin	1.02	0.68-1.52	0.89	0.57-.139	0.99	0.63-1.54
Race						
Black/African American	0.87	0.67-1.13	0.69	0.47-1.01	**0.64^c^**	**0.46-0.89**
American Indian/Native American	0.72	0.33-1.60	0.62	0.23-1.65	0.52	0.22-1.20
Asian/Pacific Islander	0.93	0.50-1.74	1.48	0.78-2.78	1.38	0.73-2.61
Other	1.44	0.77-2.71	1.44	0.73-2.85	1.42	0.72-2.79
Mother's Highest Level of Education						
> 8^th ^grade, not HS grad	1.37	0.75-2.49	1.08	0.59-1.98	1.57	0.91-2.74
Business/trade, not HS	0.77	0.16-3.58	2.04	0.40-10.48	1.74	0.39-7.85
HS graduate	0.86	0.44-1.68	0.95	0.53-1.73	1.23	0.72-2.10
Completed a GED	0.64	0.27-.155	0.62	0.27-1.41	0.87	0.41-1.81
Business/trade after HS	0.97	0.48-1.97	1.01	0.47-2.17	1.16	0.59-2.28
Non-college graduate	0.88	0.46-1.68	1.04	0.55-1.98	1.32	0.75-2.33
College graduate	0.81	0.41-1.63	0.74	0.36-1.51	1.07	0.57-2.00
Post-graduate	0.62	0.30-1.31	0.57	0.29-1.13	0.87	0.47-1.62
Total Household Income	1.00	0.99-1.001	1.00	0.99-1.001	1.00	0.99-1.001
Actual Weight/BMI						
Overweight			**1.82^c^**	**1.15-2.88**		
Obese			**4.14^c^**	**2.30-7.46**		
Weight Perception (WP)						
Slightly Overweight			**13.10^c^**	**9.59-17.88**		
Very Overweight			**23.75^c^**	**7.68-73.42**		
WP Accuracy						
Overweight or Obese, Not Overweight WP					**1.84^c^**	**1.15-2.94**
Overweight or Obese, Slightly Overweight WP					**13.59^c^**	**8.51-21.69**
Overweight or Obese, Very Overweight WP					**22.62^c^**	**7.30-70.07**

*Note*. ^a ^OR = odds ratio; ^b ^CI = confidence interval. ^c ^p < 0.05.

Wald statistics indicated that, compared to the overweight subjects, the obese subjects were significantly more likely to be trying to lose weight (*F *= 6.72, *p *< 0.0006), with no difference between a weight perception of slightly overweight and very overweight (*F *= -0.96, *p *0.392). In addition, compared to the variable of BMI, the variable of weight perception was significantly associated with greater odds of trying to lose weight; Wald statistics were significant (*p *< 0.05) for the difference between these two variables. All weight perception accuracy variables were also positively associated with trying to lose weight. Overweight or obese subjects who accurately perceived their weight as either slightly or very overweight were significantly more likely to be trying to lose weight than overweight or obese subjects who inaccurately underestimated their weight as not overweight (*F *= 38.94, *F *= 17.98, respectively *p *= 0.000). Although subjects who were overweight or obese and perceived themselves as overweight were the most likely to be trying to lose weight (*OR *= 22.62), no significant difference emerged between these subjects and those who were overweight or obese and perceived themselves as slightly overweight (*F *= 0.72, *p *= 0.3690).

#### Engaging in Exercise as a Weight-Loss Method

Among the subjects who were currently trying to lose weight (46.95%), the majority reported engaging in exercise as a weight-loss method (76.54%) (see Table 3). However, BMI, weight perception, and weight perception accuracy were not associated with the odds of engaging in exercise as a weight-loss method. Across all of the study's models, only the maternal educational attainment category of completing a General Equivalency Diploma was significantly associated with the likeliness of engaging in exercise as a weight-loss method (see Table 6). However, interpretation of this significance may be considered inappropriate because the General Equivalency Diploma category was one of multiple categories for maternal educational attainment that consisted of a small proportion of subjects (4.75%).

**Table 6:** Results of Survey-Weighted Logistic Regression for Engaging in Exercise as a Weight-Loss Methods

	Model 1		Model 2		Model 3	
			
Variable	OR^a ^	CI (95%)^b^	OR^a ^	CI (95%)^b^	OR^a ^	CI (95%)^b^
Age	0.90	0.79-1.01	0.96	0.84-1.09	0.95	0.83-1.08
Hispanic origin	0.84	0.36-1.95	0.77	0.33-1.82	0.80	0.35-1.85
Race						
Black/African American	0.75	0.37-1.54	0.73	0.36-1.47	0.76	0.36-1.58
Native American	1.53	0.47-4.99	1.50	0.45-5.00	1.49	0.41-5.37
Asian/Pacific Islander	0.45	0.14-1.46	0.41	0.12-1.37	0.43	0.13-1.45
Other	1.71	0.76-3.85	**2.22^c^**	**1.03-4.79**	**2.26^c^**	**1.05-4.88**
Mother's Education						
> 8^th ^grade, not HS grad	0.58	0.25-1.32	0.53	0.22-1.28	0.52	0.21-1.32
Business/trade, not HS	0.52	0.05-5.28	0.39	0.04-4.07	0.39	0.04-4.03
HS graduate	0.87	0.36-2.14	0.81	0.30-2.17	0.81	0.29-2.25
Completed a GED	0.34^c^	0.11-0.99	0.28^c^	0.08-0.93	0.30	0.09-1.02
Business/trade after HS	1.27	0.46-3.48	1.00	0.34-2.96	1.01	0.33-3.10
Non-college graduate	0.98	0.39-2.45	0.88	0.32-2.40	0.93	0.32-2.64
College graduate	1.13	0.43-3.00	0.92	0.32-2.63	0.92	0.31-2.73
Post-graduate	0.90	0.28-2.88	0.70	0.21-2.30	0.66	0.19-2.32
Total Household Income	1.01	0.99-1.01	1.01	0.99-1.01	1.01	0.99-1.01
Actual Weight/BMI						
Overweight			1.15	0.68-1.93		
Obese			0.75	0.44-1.28		
Weight Perception (WP)						
Slightly Overweight			1.01	0.63-1.61		
Very Overweight			0.96	0.43-2.14		
WP Accuracy						
Overweight or Obese, Not Overweight WP					0.58	0.25-1.35
Overweight or Obese, Slightly Overweight WP					0.93	0.59-1.45
Overweight or Obese, Very Overweight Weight WP					1.23	0.52-2.93

*Note*. ^a ^OR = odds ratio; ^b ^CI = confidence interval. ^c ^p < 0.05.

## Discussion

Body-mass index, weight perception, and weight perception accuracy were positively associated with trying to lose weight in the study's representative sample of U.S. female adolescents, after controlling for selected covariates. Overweight or obese female adolescents in the study were more likely to be trying to lose weight than those who were not overweight or obese, a finding that is consistent with previous studies of U.S. [16,22,27] and international [20,23] adolescents. In addition, the obese female adolescents in the current study were significantly more likely to be trying to lose weight than those who were overweight, another finding consistent with previous research on international adolescents [22]. Because the likeliness of the obese female adolescents in the present sample to be trying to lose weight was greater (4.14 times more likely than other female adolescents) than the likeliness of their international counterparts (1.95 times more likely) [22], it is plausible that obese U.S. female adolescents are more likely to be trying to lose weight than obese female adolescents in other countries.

Results from the current study also demonstrated that U.S. female adolescents who perceive themselves as slightly or very overweight are significantly more likely to be trying to lose weight than are those who do not perceive themselves as overweight, with no difference in the likeliness of trying to lose weight between a weight perception of slightly overweight and very overweight. This finding is comparable to a previous study of adolescents in Beirut [23] and addresses the lack of studies considering such an association in a sample of U.S. female adolescents.

The current study's finding that weight perception has a stronger association than BMI with trying to lose weight may raise concerns, because the association between weight perception and trying to lose weight is often related to eating disorders. However, such concerns may be assuaged by the finding that overweight or obese female adolescents with an accurate weight perception are more likely to be trying to lose weight than those who inaccurately perceive their weight. Furthermore, the study's overweight or obese female adolescents who underestimated their weight (20%) were significantly less likely to be trying to lose weight than those who accurately perceived their weight as either slightly or very overweight. Further studies are needed to determine the consistency of these findings among both U.S. and international female adolescents. In addition, researchers and clinicians who design weight-loss interventions for female adolescents are encouraged to consider weight perception accuracy, not just BMI or weight perception.

Additional studies with longitudinal designs are also needed to determine the validity of the association between age and efforts to lose weight among female adolescents. Results from the current study demonstrated older female adolescents are more likely than their younger counterparts to be trying to lose weight, a finding that is consistent with previous research [22,26]. However, studies have also demonstrated negative [42] and nonsignificant [25] associations between adolescents' age and efforts to lose weight. Consistent findings from future research will allow clinicians to identify whether weight-loss interventions should be aimed at older, younger, or all female adolescents.

Weight-loss interventions targeted towards or designed specifically for Black/African American U.S. female adolescents may also be needed. Black/African American female adolescents in the study had a relatively high prevalence of overweight and obesity (38.19%) and a low prevalence of perceiving themselves as slightly or very overweight (36.94%). Also, compared to the other race categories in this study sample, Black/African American female adolescents who were overweight or obese were the most likely to inaccurately underestimate their weight (10.74%), and they were significantly less likely than their White counterparts to be trying to lose weight.

This study identified significant associations between BMI, weight perception, and weight perception accuracy with trying to lose weight. In addition, a relatively large number of this study's sample reported engaging in exercise as a weight-loss method (*n *= 796). Thus it is surprising that these variables were not associated with engaging in exercise as a weight-loss method. It is possible that female adolescents may engage in other healthy, unhealthy, or extreme weight-loss behaviours in addition to or in place of exercise. Including additional weight-loss behaviours in this study's regression models may have strengthened the model, possibly allowing for significant associations. Thus, additional healthy and unhealthy weight-loss behaviours are recommended in future research on the associations of BMI, weight perception, and weight perception accuracy with engaging in exercise as a weight-loss method among overweight and obese female adolescents.

## Limitations

Use of the Add Health data [30] in this secondary-analysis study limited the statistical analyses of data and the generalizability of the study's findings. The Add Health sample was selected from a school-based sample; thus, findings are only generalizable to U.S. female adolescents enrolled in school. Furthermore, the underlying survey design of the Add Health study did not allow for inferences of cause and effect. Although a well-powered study sample with data from more than 2,000 subjects was available for analysis, there was a relatively small number of subjects who were overweight or obese, which did not allow for separate analyses for overweight and obese subjects. The weight perception accuracy variable, therefore, combined subjects who were overweight with those who were obese, and statistical analysis was unable to consider differences in weight perception accuracy between overweight and obese subjects. Also, the conceptualization of weight perception accuracy and the subjects' interpretation could have affected their responses, making it difficult to "match" the BMI categories of overweight and obese with the weight perception categories of slightly overweight and very overweight. In terms of validity and reliability, there was a lack of reported measures, and recommended techniques were not available for this secondary-analysis study.

## Conclusions

Based on the findings of this secondary analysis of data from the Add Health study [30], BMI, weight perception, and weight perception accuracy are significantly positively associated with the likeliness of trying to lose weight among U.S. female adolescents. However, BMI, weight perception, and weight perception accuracy are not associated with the likeliness of engaging in exercise as a weight-loss method among U.S. female adolescents; nevertheless, this finding does not indicate that such associations do not exist. Considering the strengths and limitations of this study, results should be interpreted with a degree of caution. Although this study's results support findings from previous research regarding adolescents' efforts to lose weight, the current investigation is the first known study to consider the association of weight perception accuracy with trying to lose weight and engaging in exercise as a weight-loss method among overweight or obese U.S. female adolescents. To help reduce the national and worldwide epidemic of overweight and obesity among female adolescents, weight perception accuracy, as well as age and race, must be considered as significant factors associated with weight-loss efforts among female adolescents.

## Competing interests

The authors declare that they have no competing interests.

## Authors' contributions

JY had full access to all the data in the study and takes responsibility for the integrity of the data and the accuracy of the data analysis, including interpretation, as well as drafted and critically revised the manuscript. BKM contributed to the design of the study and revisions of the manuscript. WB contributed to the design of the study, interpretation of the data, and revisions of the manuscript. RN contributed to the design of the study and data analysis and interpretation, as well as revisions of the manuscript. All authors read and approved the final manuscript.

## Pre-publication history

The pre-publication history for this paper can be accessed here:

http://www.biomedcentral.com/1471-2458/10/465/prepub
